# Lifestyle factors and plasma biomarkers of Alzheimer's disease: A narrative review

**DOI:** 10.1016/j.tjpad.2025.100130

**Published:** 2025-03-12

**Authors:** Claudie Hooper, Nicola Coley, Julien Delrieu, Sophie Guyonnet

**Affiliations:** a IHU HealthAge, Gérontopôle, Department of Geriatrics, CHU Toulouse, Toulouse, France; b Aging Research Team, Centre for Epidemiology and Research in Population health (CERPOP), INSERM-University of Toulouse, 37 allées Jules Guesde, 31073 Toulouse, Toulouse, France; c Department of Epidemiology and Public Health, Toulouse University Hospital, 37 allées Jules Guesde, 31073 Toulouse, Toulouse, France; d IHU HealthAge, Cité de la santé, place Lange, 31059 Toulouse, France; e CERPOP Inserm UMR 1295, Toulouse, France. University of Toulouse, Toulouse, France

**Keywords:** Alzheimer's disease, Plasma, Biomarkers, Lifestyle, INSPIRE

## Abstract

Alzheimer's disease (AD) is a neurodegenerative disorder characterised by amyloid-β (Aβ), tau hyperphosphorylation and neurodegeneration. Blood-based biomarkers are emerging as a minimally invasive tool for disease detection and monitoring. This review depicts the relationships between modifiable lifestyle factors (nutrition, physical activity (PA), sleep, alcohol consumption, smoking, and social isolation) and plasma biomarkers of AD: Aβ_42_, Aβ_40_, Aβ_42/40,_ phosphorylated tau, total tau, neurofilament light chain (NfL) and glial fibrillary acidic protein. Limited evidence suggests that better nutrition is associated with favourable AD plasma biomarker profiles and that PA is associated with less plasma NfL and Aβ, whilst poor sleep is associated with elevated plasma Aβ. However, lack of data and inconsistent findings highlight the need for further investigation to substantiate or refute these trends. Moreover, future research should include the analysis of lifestyle on plasma biomarkers according to gender, metabolic health and *APOE* status. Considering the growing emphasis on modifiable lifestyle factors for preventing and delaying dementia onset further investigation is justified.

## Highlights

• Relationships between lifestyle factors and plasma biomarkers of Alzheimer's disease.
• Better nutrition is associated with favourable Alzheimer's plasma biomarker profiles.
• Exercise is associated with less plasma neurofilament light chain and β-amyloid.
• Poor sleep is associated with elevated plasma β-amyloid.
• Lack of data and inconsistent findings highlight the need for further research.

## 1 Background

Alzheimer's disease (AD) is a progressive neurodegenerative disorder characterised by reduced cognition, particularly memory, as well as behavioural disturbances [1]. The amyloid cascade hypothesis more than 30 years after its first proposal still provides the leading theory to explain disease manifestation. According to this hypothesis, amyloid-β (Aβ), which deposits in the form of plaques, leads to tau hyperphosphorylation and in turn the formation of neurofibrillary tangles (NFTs) with ensuing neurodegeneration causing the cognitive symptoms of AD [2,3]. Aβ is therefore considered the causal agent of AD precipitating down-stream events. The National Institute on Aging and Alzheimer's Association (NIA-AA) has classified or staged AD according to the presence of amyloid (A), tau pathology (T) and neurodegeneration (N) giving rise to the ATN framework [4]. This framework is based on the findings of brain imaging and cerebrospinal fluid (CSF) assessment of pathology. However, the high cost of brain imaging and the invasiveness and requirement for a skilled clinician to perform lumbar puncture coupled with the advent of ultra-sensitive assay techniques has fuelled research into the characterisation of blood-based ATN biomarkers [5,6].

In accordance with the ATN framework, Aβ_42_ and the Aβ_42/40_ ratio are decreased in the blood in AD [6]. Conversely, the abundance of Aβ_40_ is typically unchanged until late in disease [7] and is therefore used to normalise Aβ_42_ for inter-individual differences [5,8]. The specific reduction in blood-based Aβ_42_ is thought to be due to its oligomerisation and sequestration into plaques in the brain [8]. A major limitation of the plasma Aβ_42/40_ ratio is that its levels only decrease about 10–20 % in individuals with cerebral Aβ pathology (compared to an approximate 40–60 % reduction in CSF), which therefore results in a reduction of assay sensitivity [5,6]. Note, a lower plasma Aβ_42/40_ ratio can be due to a decrease in plasma Aβ_42_ or an increase in plasma Aβ_40_ or a reduction in both species.

Arguably, isoforms of phosphorylated tau (p-tau) such as p-tau threonine 181, 231 and 217 in the blood, which correlate with cerebral tauopathy and cerebral Aβ positivity, provide more robust biomarkers of AD exhibiting large fold-changes in plasma in disease [5,6,8]. Blood-borne neurofilament light chain (NfL) and to a lesser extent total tau levels provide a direct measure of neurodegeneration, but are non-specific for AD [5,9]. Glial fibrillary acidic protein (GFAP), a marker of astrocyte activation and hence neuroinflammation is often measured in combination with the aforementioned ATN biomarkers [10]. Plasma GFAP is also predictive of central Aβ pathology and future cognitive decline possibly due to its plaque-association [11].

In this narrative review, we summarise the emerging body of literature in humans describing the associations and effects of nutrition, physical activity (PA), alcohol consumption, smoking and social isolation on ATN plasma biomarkers of AD (including GFAP). The importance of such modifiable risk factors for preventing and delaying the onset of dementia has been highlighted in the recent Lancet Commissions on dementia prevention, intervention, and care [12,13]. Thus, a better understanding of the relationships between such modifiable lifestyle factors and plasma biomarkers of AD is timely and essential to clarify how lifestyle influences underlying disease mechanisms and disease course. This in turn could inform public health guidelines leading to personalised lifestyle plans for early intervention in individuals at risk of AD.

## 2 Methods

A narrative literature review was undertaken using PubMed, Embase, and Web of sciences databases to provide an overview of the relationships between lifestyle factors and plasma biomarkers of AD. To extract relevant manuscripts a Boolean search strategy was employed as follows: (“diet” OR “dietary patterns” OR “dietary factors” OR “food” OR “nutrition” OR “sleep” OR “fatigue” OR “nicotine” OR “alcohol” OR “physical activity” OR “exercise” OR “social isolation” OR “loneliness”) AND (“tau” OR “p-tau” OR “t-tau” OR “Aβ42” OR “Aβ40” OR “Aβ” OR “βA” OR “amyloid” OR “β amyloid” OR “β-amyloid” OR “amyloid-β” OR “amyloid β“ OR “NFL“ OR “neurofilament light chain“ OR “GFAP“ OR “glial fibrillary acidic protein“) AND (“plasma”) AND (“Alzheimer*” OR “cognit*”). The search was limited to the ‘Title/Abstract’ domain in PubMed and Embase and the ‘Abstract’ domain in Web of Science. No date limits were set for the search. The literature search was performed in October 2024. Only original research articles published in journals and written in English were included. Titles retrieved were subjected to suitability screening through the assessment of relevance by one author. Cross-sectional, longitudinal, and interventional studies in humans were included that reported on the following plasma biomarkers: Aβ_42_, Aβ_40_, Aβ_42/40_, p-tau, total-tau, NfL or GFAP (no strict inclusion criteria for cognitive status was set: studies included participants with cognition ranging from normal through to severe Alzheimer's disease). Studies investigated whether these biomarkers varied according to lifestyle factors, limited for the purpose of this review to, nutrition, PA, alcohol consumption, smoking or social isolation. Manuscripts reporting positive as well as negative findings were included in the review and there were no exclusion criteria due to the paucity of publications.

## 3 Results: description of the main findings from the studies

Thirty-nine articles were retrieved for inclusion in this narrative review. Ten studies were included on nutrition (summarised in Table 1), 12 studies on PA (summarised in Table 2), 14 studies on sleep (summarised in Table 3), 2 studies on alcohol, one article on smoking and one study on social isolation (summarised in Table 4).

**Table 1:** Summary of studies reporting associations between nutrition and plasma biomarkers of Alzheimer's disease.

Reference	Study (country)	Age, n, gender, cognitive information	Lifestyle factor	Plasma biomarkers investigated and method used	Main findings relating to plasma biomarkers
Gu et al., 2012 [14]	Washington Heights/Hamilton Heights Columbia Aging Project (WHICAP). US. Cross-sectional study.	75.4 ± 6.1 years, *n* = 1219, 68 % female Cognitively healthy.	**Nutrition**: Saturated fatty acids, monounsaturated fatty acids, omega-3 polyunsaturated fatty acids (PUFAs), omega-6 PUFAs, vitamin E, vitamin C, β-carotene, vitamin B(12), folate and vitamin D. Measured using 61-item version of Willett's Semiquantitative Food Frequency Questionnaire (SFFQ).	Aβ_40_ and Aβ_42_ (sandwich ELISA).	↑ n-3 PUFA ↓ Aβ_42._
Giudici et al., 2023 [16]	Multidomain Alzheimer Preventive Trial (MAPT). France. Cross-sectional study.	76.8 ± 4.5 years, *n* = 475, 59.4 % female.	**Nutrition**: Erythrocyte docosahexaenoic acid, eicosapentaenoic acid and omega-3 index, plasma homocysteine and plasma 25 hydroxyvitamin D.	Aβ _42/40_ and NfL (Aβ: immunoprecipitation followed by LCMS and NfL: Meso Scale immuno-assay).	↑ homocysteine ↑ NfL.
Duggan et al., 2023 [18]	Women's Health Initiative Memory Study (WHIMS). US. Cross-sectional study.	71.3 ± 3.8 years, *n* = 1528 100 % female Without cognitive impairment.	**Nutrition**: Inflammatory diet proteins identified with proteomics.	Aβ _42/40_ and NfL (SiMoA).	↑ CXCL10, CCL3, CDCP1, OPG ↑ NfL ↑ CXCL10, CCL3, NFATC3, HGF, OPG ↑ Aβ_42/40._
Chu et al., 2024 [19]	Shanghai Sixth People's Hospital Affiliated to Shanghai Jiao Tong University School of Medicine. China Cross sectional study.	65.2 ± 6.9 years, *n* = 129, 55 % female Cognitively unimpaired.	**Nutrition:** Mini-Nutritional Assessment Short-Form (MNA-SF).	Aβ_42_, Aβ_40_, Aβ _42/40,_ total-tau, p-tau-181 and NfL (SiMoA).	↑ MNA-SF ↑ Aβ_42/40_ and ↑ MNA-SF and normal nutrition ↓ p-tau-181 in Aβ-PET(+) participants.
Gentreau et al., 2022 [20]	Three-City (3C) cohort. France. Cross-sectional study.	76.1 ± 5.2 years, *n* = 377 60.2 % female.	**Nutrition:** Glycaemic load determined using data from the 148-item semi-quantitative Food Frequency Questionnaire.	Aβ_42,_ Aβ_40,_ Aβ_42_/Aβ_40_ (INNO-BIA Kit immunoassay).	↑ Lunch GL ↓ Aβ_42_ and ↓ Aβ_42_/Aβ_40_.
Huber et al., 2023 [21]	Institute of Nutrition and Food Sciences, University Bonn. Germany. Cross-sectional study.	60 ± 7 years, *n* = 111, 58 % female Cognitively unimpaired.	**Nutrition:** Test meal in the form of a protein chocolate drink (300ml: 303.8 kcal; carbohydrates 41.8 g of which 19 g sugar; protein 19.0 g; fat 7.6 g; Boost High Protein, Nestlé Health Science, Vevey, Switzerland).	NfL, GFAP, Aβ_42,_ Aβ_40,_ Aβ_42/40_, p-tau 181, p-tau 231, and total-tau. (SiMoA).	Significant differences were found for NfL, GFAP, Aβ_42/40_, p-tau 181, and p-tau 231 between the fasting group and the postprandial group. The greatest change to baseline occurred for GFAP and p-tau 181 (120 min postprandially.
Fernando et al., 2018 [22]	Australian Imaging, Biomarkers and Lifestyle (AIBL) study of aging. Australia. Cross-sectional study.	69.8 ± 6.8 years, *n* = 541 59 % female Cognitively normal.	**Nutrition:** Dietary intakes of protein and fibre assessed using the Cancer Council of Victoria Food Frequency Questionnaire (CCVFFQ).	Aβ _40_, Aβ_42,_ Aβ_42/40_ (INNO-BIA plasma Aβ forms assays)	No associations were found between intake of protein, fibre, or the protein to fibre intake ratio and Aβ_40_, Aβ_42_ or Aβ_42/40._
Chen et al., 2016 [23]	TFA-AD trial, Neurology Central Hospital of Tianjin. China. RCT.	Age > 55 years *n* = 121 50 % female AD: MMSE total score between 3 and 26 on donepezil.	**Nutrition:** Folic acid (1.25 mg/d) for 6 months.	Aβ _40_, Aβ_42,_ Aβ_42/40_ (ELISA).	Folic acid significantly ↓ Aβ_40_ in the intervention group compared to placebo.
Giudici et al., 2023 [24]	The Nolan Study 18 centres in France. Secondary analysis of an RCT.	78.1 ± 4.7 years, *n* = 289 56.4 % female MMSE ≥ 24.	**Nutrition**: Daily nutritional blend for one year: 50 mg thiamin, 15 mg riboflavin, 25 mg niacin, 23 mg pantothenic acid, 18 mg pyridoxine, 0.15 mg biotin, 0.4 mg folic acid, 0.5 mg cobalamin, 82.6 mg vit E, 500 mg vit C, 15μg vit D, 85 mg choline, 80μg selenium, 3*g* citrulline, 700 mg eicosapentaenoic acid and 770 mg docosahexaenoic acid.	p-tau 181 and GFAP (SiMoA).	No intervention effect on p-tau 181 and GFAP.
Uchiyama-Tanaka et al., 2024 [25]	Soushinkai, a daycare rehabilitation facility in Okayama. Japan. RCT.	79.0 ± 8.8 years *n* = 43 60 % female Without cognitive dysfunction.	**Nutrition**: Group receiving dietary guidance to reduce advanced glycation end products and a group receiving dietary guidance + macrophage-activating factor.	Aβ_42/40_ (immunoprecipitation followed by LCMS).	No significant differences in serum Aβ_42/40_ between the 2 intervention groups and placebo.
Coley et al., 2024 [26]	Multidomain Alzheimer Preventive Trial (MAPT). France. Secondary analysis of an RCT.	Median age: 74 years, IQR: 72–78, *n* = 527 67 % female 40 % CDR of 0.5. Intervention duration: 3 years.	3 groups: multidomain intervention comprising cognitive training, advice on physical activity and nutrition with or without omega-3 fatty acid supplementation (800 mg docosahexaenoic acid and 225 mg eicosapentaenoic acid daily) plus omega-3 fatty acid supplementation alone.	p-tau 181 (SiMoA).	No significant differences in change in p-tau 181 between the placebo and the 3 intervention groups.

**Abbreviations**: Aβ; amyloid-β, GFAP; ELISA; Enzyme-Linked Immunosorbent Assay, GFAP; Glial Fibrillary Acid Protein, LC-MS; liquid chromatography - mass spectrometry, MMSE; Mini Mental State Examination, NfL; Neurofilament Light, RCT; randomised controlled trial, SiMoA; Single Molecule Array, vit; vitamin.

**Table 2:** Summary of studies reporting associations between physical activity and plasma biomarkers of Alzheimer's disease.

Reference	Study (country)	Age, n, gender, cognitive information	Lifestyle factor	Plasma biomarkers investigated and method used	Main findings relating to plasma biomarkers
Guo et al., 2024 [27]	Chinese preclinical Alzheimer's disease study (C-PAS). China. Cross-sectional study.	67.9 ± 11.5 years total population, *n* = 1048 62 % female total population MMSE: 22.9 ± 7 total population.	**Physical activity:** Maximum grip strength test and timed up and go test as a measure of physical function.	Aβ_42/40_ and NfL (no method specified).	↑ Physical function ↓ NfL.
Wang et al., 2023 [28]	Multimodal INterventions to delay Dementia and disability in rural China (MIND-China), China. Cross-sectional study.	69.56 ± 4.47 years, *n* = 1029 59.48 % female Dementia-free.	**Physical activity**: Accelerometer measured SB, LPA and MVPA as < 100, 100–1040, and ≥ 1041 CPM.	NfL (SiMoA).	↑ daily SB time or ↓daily LPA and MVPA ↑ NfL: when SB time ≥ 8.00 h/day or LPA time < 5.00 h/day or MVPA time < 2.00 h/day.
Raffin et al., 2021 [29]	Multidomain Alzheimer Preventive Trial (MAPT). France. Cross-sectional study.	76.8 ± 4.5 years, *n* = 465 40.0 % female Dementia-free at risk of cognitive decline.	**Physical activity:** Short form of the Minnesota Leisure Time Activities questionnaire used to calculate metabolic equivalent task (MET) minutes per week (MET-min/week).	Aβ _42/40_ and NfL (Aβ: immunoprecipitation followed by LCMS and NfL: Meso Scale immuno-assay).	500–999 metabolic equivalent task (MET)-min/week) and 2000+ MET-min/week of PA ↓ (ln)NfL compared to control. Performing > 90 MET-min/week of PA ↓ probability of ↑ plasma NfL. No association of PA with Aβ_42/40._
He et al., 2020 [30]	Multidomain Alzheimer Preventive Trial (MAPT). France. Cross-sectional and longitudinal study.	76 ± 5 years, *n* = 452 59 % female MMSE: 27.8 ± 1.9.	**Physical activity:** Physical function was evaluated as gait speed, which was measured by a 4-m usual-pace walk test (m/s), and chair-stand time (s), which was measured by a 5-repetition maximal speed chair-stand test.	Aβ _42/40_ and NfL (Aβ: immunoprecipitation followed by LCMS and NfL: Meso Scale immuno-assay).	No association between Aβ+NfL+ in plasma and physical function (gait speed and chair stand time) cross-sectionally. Aβ+NfL+ participants had ↑ annual declines in gait speed when *APOE* ε4 was excluded from the mixed effect-linear model.
Brown et al., 2013 [31]	Australian Imaging, Biomarkers and Lifestyle (AIBL): Australia. Cross-sectional study.	69.6 ± 6.8 years, *n* = 546 57 % female Cognitively normal.	**Physical activity:** International Physical Activity Questionnaire (IPAQ).	Aβ _40_, Aβ_42,_ Aβ_42/40_ (INNO-BIA Kit immunoassay and Mehta ELISA).	↑PA ↓Aβ_42/40_ in *APOE* ε4 non-carriers.
Pedrini et al., 2022 [32]	Australian Imaging, Biomarkers and Lifestyle (AIBL): Australia. Cross-sectional study.	74.0 ± 5.5 *n* = 143, 46.2 % female Cognitively normal: MMSE ≥ 25.	**Physical activity**: International Physical Activity Questionnaire (IPAQ).	Aβ _40_, Aβ_42,_ Aβ_40/42_ (immunoprecipitation + MALDI-TOF mass spectrometry).	↑PA ↓Aβ_40_ and Aβ_42_, in *APOE* ε4 noncarriers and in Aβ-PET(-) participants.
Stillman et al., 2017 [33]	Cardiovascular Health Study (CHS) – cognition study. US. Longitudinal study.	79.51 ± 3.15 years, *n* = 149, 72 % female, Cognitively normal.	**Physical activity:** Modified Minnesota Leisure‐Time Activities Questionnaire (MLTAQ).	Aβ _40_, Aβ_42,_ Aβ_42/40_ (sandwich ELISA).	↑ PA at baseline ↓ Aβ_42_ 9 and 13 years later and ↓ Aβ_42/40_ 13 years on.
Yoon et al., 2023 [34]	Kasama Study health check in 2019. Japan. Cross-sectional study.	age: ≥ 65 years, *n* = 325, 67.1 % female Dementia-free.	**Physical activity**: The dual task exercise group, included individuals regularly practicing square-stepping exercise at least once a week for >1 year. The single task exercise group, included individuals performing daily exercise, such as walking, general fitness exercise, golf, and dance for ≥30 min continuously, at least twice a week for >1 year.	Aβ_42_ (sandwich ELISA).	↑ Aβ_42_ in the dual task exercise group than in the non-exercise group.
Yu et al., 2022 [35]	FIT-AD trial. US Pilot RCT.	77.6 ± 6.99 years *n* = 26 35.6 % female MMSE: 21.6 ± 3.32.	**Physical activity:** 2:1 allocation ratio to moderate-intensity cycling or low-intensity stretching for 20–50 min, 3 times a week for 6 months.	Aβ_42/40,_ p-tau 181, and total-tau (SiMoA).	Effect sizes for within-group 6-month difference were observed for plasma p-tau 181 in the group allocated to stretching (d = 0.43 [−0.33, 1.19]) and plasma total-tau in the cycling group (d =−0.35 [−0.87, 0.17]).
Yokoyama et al., 2015 [36]	Community dwellers in Sumiyoshi-ku, Osaka City, Japan, RCT.	≈74 years, *n* = 27 92 % female Modified Mini-Mental State (3MS) examination ≥ 60	**Physical activity:** Participants in the dual task (DT) and single task groups received 1-hour of exercise training 3 times a week, for 12 weeks. Training comprised 15 min of mental gymnastics, 25 min of resistance training, 10 min of aerobic exercise, and 10 min of systemic flexibility exercise. The DT group, received concurrent cognitive tasks (arithmetic or Shiritori) performed during resistance training and aerobic exercise.	Aβ _40_, Aβ_42,_ Aβ_42/40_ (ELISA)	Aβ_42_ ↓ and Aβ_40_ ↑ following the intervention in both groups, but the change in Aβ_42_ was not statistically significant in the dual task group. Aβ_42/40_ significantly ↓ in both groups following the training, although there was no significant difference between groups in Aβ_42/40._
Sewell et al., 2024 [37]	Intense Physical Activity and Cognition (IPAC) study. New Zealand. RCT.	69.1 ± 5.2 years *n* = 99, 55 % female MMSE > 27.	**Physical activity:** Moderate and high intensity groups cycled on an ergometer (Wattbike Pro; Wattbike, Australia) for 50 min, twice a week for 6 months. The high intensity exercise group completed a 10-min warm up, 11 intervals of 1 min cycling at hard exertion at 18.0 Borg Scale, 80 % aerobic capacity, interspersed with 2 min of active recovery, and a 9-min cool down. The moderate intensity group cycled at a constant intensity for 50 min (50–60 % aerobic capacity; 13.0 Borg Scale). The control group received a 2-hour session on exercise benefits.	Aβ_42,_ Aβ_40_, Aβ_42/40_, p-tau 181, GFAP and NfL (SiMoA).	No change in p-tau 181, Aβ_40_, Aβ_42_, Aβ_42/40,_ GFAP, or NfL pre- to post-intervention.
Baker et al., 2010 [38]	University of Washington School of Medicine. USA. RCT.	≈ 70 years *n* = 28 64.3 % female Cognitively normal with prediabetes or newly diagnosed type 2 diabetes mellitus	**Physical activity:** Participants in both groups carried out activity routines 4 day/week for 45–60 min/session for 6 months. In the aerobic group, exercise duration and intensity were titrated up over the first 6 weeks, until participants were exercising at 75–85 % of heart rate reserve using a treadmill, stationary bicycle, or elliptical trainer. This intensity was maintained for the study duration. The control group carried out stretching and balance exercises, maintaining heart rate ≤ 50 % HR reserve.	Aβ_42,_ Aβ_40_ (ELISA)	In exploratory analysis examining the mechanism of the effects of PA on cognition, there was found to be a non-significant trend towards ↓ Aβ_42_ (*p* = 0.07) in the aerobic exercise group relative to the control group.

**Abbreviations**: Aβ; amyloid-β, SB; Sedentary Behaviour, CPM; Counts per minute, DEG; dual task exercise group, ELISA; Enzyme-Linked Immunosorbent Assay, eGFAP; Glial Fibrillary Acid Protein, LC-MS; liquid chromatography - mass spectrometry, LPA; light-intensity physical activity, MET; metabolic equivalent task, MMSE; Mini Mental State Examination, MVPA; moderate-to-vigorous-intensity physical activity, NfL; Neurofilament Light Chain, NEG; non-exercise group, PA; physical activity, RCT; randomised Controlled trial, SEG; single task exercise group, SiMoA; Single Molecule Array.

**Table 3:** Summary of studies reporting associations between sleep metrics and plasma biomarkers of Alzheimer's disease.

Reference	Study (country)	Age, n, gender, cognitive information	Lifestyle factor	Plasma biomarkers investigated and method used	Main findings relating to plasma biomarkers
Tang et al., 2024 [39]	Multimodal Interventions to Delay Dementia and Disability in Rural China (MIND-China) study. China. Cross-sectional study.	age ≥ 60 year, *n* = 841 Dementia-free.	**Sleep:** Electrocardiogram-based cardiopulmonary coupling analysis to measure sleep duration.	Aβ_40_, Aβ_42_, Aβ_42/40_ total-tau and NfL (SiMoA).	Long sleep duration (> 8 h versus 6–8 h) measured objectively ↑ plasma Aβ_40_ and total-tau and ↓ Aβ_42_/Aβ_40._
Baril et al., 2024 [42]	Presymptomatic Evaluation of Experimental or Novel Treatments for AD (PREVENT‐AD) cohort. Canada. Cross-sectional study.	68.25 ± 5.41 years, *n* = 203 38.4 % female Dementia-free.	**Sleep:** Actigraphy over 6–7 nights was used to measure sleep midpoint, sleep duration (measure of sleep quantity), sleep efficiency (measure of sleep quality) and average activity count per minute within each individual's sleep period (measure of sleep fragmentation).	Aβ_42,_ Aβ_42/40_ (immunoprecipitation followed by LC-MS) p‐tau181 and p‐tau 231 (SiMoA) Ratios other than Aβ_42/40_ used: p‐tau 181/Aβ_42_ p‐tau 231/Aβ_42_	↑ variability of both objectively-measured sleep duration and night-time activity count ↑ p-tau 231/Aβ_42_. ↑ p‐tau 181 and p‐tau 231 ↑ sleep duration variability and ↑ p‐tau 231 ↑ night-time activity count variability.
Gao et al., 2021 [43]	Participants from the suburbs of Xi'an, China. Approved by the First Affiliated Hospital of Xi'an Jiao Tong University. Cross-sectional study.	57.4 ± 9.7 years, *n* = 1459 59 % female Cognitively normal.	**Sleep**: The Pittsburgh Sleep Quality Index (PSQI). PSQI score: 〈5 for good sleep quality, scores of 6–10 for general sleep quality, and scores of 〉 10 for poor sleep quality.	Aβ_40_ and Aβ_42,_ Aβ_42/40_ (ELISA).	↑ Sleep quality ↓ log-transformed Aβ_40._ ↑ PSQI score ↑ log-transformed Aβ_40_ and ↓ Aβ_42_/Aβ_40._ Log-transformed Aβ_42_ no correlation with sleep quality or the PSQI score.
Chu et al., 2023 [44]	Shanghai Sixth People's Hospital Affiliated to Shanghai Jiao Tong University School. China. Retrospective study with respect to Aβ PET imaging with cross-sectional design for the Sleep/biomarker analysis.	64.4 ± 7.8 years, *n* = 335 62.4 % female MMSE > 24 for > 6 years of education, > 20 for 1–6 years of education.	**Sleep:** The Pittsburgh Sleep Quality Index (PSQI).	Aβ_42_, Aβ_40_, Aβ_42/40_, total-tau, p-tau 181, NfL. (SiMoA).	In Aβ-PET(+) adults falling asleep at ≥ 22:00 to ≤ 23:00 ↑ Aβ_42_ and ↑ Aβ_42/40_. ↑Aβ_42/40_ ↑ sleep efficiency value, no/mild daytime dysfunction and PSQI score ≤ 5 (good sleep quality). ↑ p-tau-181 was associated with sleep latency > 30 min in Aβ-PET(+) adults and moderate/severe sleep disturbance in Aβ-PET(-) adults.
Liu et al., (2023) [45]	Cognitive Disorders Clinics in the First People's Hospital of Foshan. China Cross-sectional study.	69.68 ± 6.84 years, *n* = 305 59.7 % female MMSE:25.83 ± 2.38.	**Sleep**: Pittsburgh Sleep Quality Index (PSQI).	Aβ_42_, Aβ_40,_ Aβ_42/40_ (sandwich ELISA).	↑ PSQI score ↑Aβ_42_ in the aMCI group, but not in the normal cognition group. ↑ PSQI score ↑Aβ_42_/Aβ_40_ in both the normal cognition and aMCI groups. The association between PSQI score and Aβ_42_/Aβ_40_ was stronger in individuals with aMCI relative to normal cognition.
Grimmer et al., 2020 [48]	University hospital Klinikum rechts der Isar, Technical University of Munich (Munich, Germany). Short term repeated measures study.	34 ± 4.3 years, *n* = 17 41 % female.	**Sleep**: Sleep duration and fragmentation measured with sleep record keeping and verified with actometers.	Aβ_42,_ Aβ_40,_ Aβ_42/40_ (ELISA).	During on-call nights, a 10.7 % reduction of Aβ_42_ occurred overnight (compared to 11.4 % on nights spent at home). Each sleep interruption ≤15 min diminished this reduction by 5.4 %.
Wei et al., 2017 [49]	The First Affiliated Hospital of Xi'an Jiao Tong University. China Single arm trial.	27.3 ± 3.4 years *n* = 20 45 % female Cognitively normal.	**Sleep**: One night of total sleep deprivation under the supervision of researchers.	Aβ_42_, Aβ_40,_ Aβ_42/40_ (ELISA).	Total sleep deprivation significantly ↑ morning Aβ_40_ by 32.6 % and ↓ Aβ_42_/Aβ_40_ by 19.3 %.
Benedict et al., 2020 [50]	Uppsala University Biomedical Center. Sweden. Cross-over study.	22.3 ± 0.5 years *n* = 15 0 % female.	**Sleep**: Normal control or sleep-deprived conditions (participants were under continued constant direct visual supervision by the experimental leaders to ensure sustained wakefulness). At least 4 weeks elapsed between the two different sessions (sleep loss versus normal sleep).	total-tau, Aβ_40_, and Aβ_42,_ NfL, GFAP (SiMoA).	In complete overnight sleep loss, the evening to morning change in total-tau ↑ compared to normal sleep (+17.2 % versus +1.8 % change). No changes between the sleep conditions were observed for Aβ_40_, Aβ_42_, NfL, or GFAP.
Liu et al., (2020) [51]	Participants from the Knight Alzheimer Disease Research Center/ volunteer registry at Washington University (Volunteers for Health). US Cross-over study.	≈ 48 years *n* = 5 60 % female Cognitively normal MMSE ≈ 28.	**Sleep**: Normal control or sleep-deprived conditions (kept awake for 36 h by nursing staff), and then returned ∼4–6 months later on average for cross-over to the other condition.	Aβ_40_, Aβ_42_, unphosphorylated tau-181 (total tau 181), unphosphorylated tau-217 (total tau 217), and p-tau 181. (immunoprecipitation followed by LC-MS).	One night of sleep deprivation (versus normal sleep) ↓ Aβ_40,_ Aβ_42_, total-tau and p-tau 181 in plasma, whilst an ↑ in these biomarkers was seen in CSF. ≈ 5–15 % ↓ plasma ≈ 35–55 % ↑ CSF
Rosenblum et al., (2024) [54]	Max Planck Institute of Psychiatry. Germany. Cross-sectional study.	39.8 ± 16.0 years *n* = 60 53.3 % female.	**Sleep:** Polysomnography.	Aβ_42_ and Aβ_40_ (ELISA).	Slow-wave oscillations (deep sleep) correlated with ↑ Aβ_40_ and Aβ_42_, whereas rapid eye movement (REM) sleep correlated with ↓Aβ_40_ and Aβ_42._
Sanchez-Espinosa et al., 2014 [55]	Pablo de Olavide University. Recruitment from older people's associations, community health screening, and outpatient services. Spain. Cross-sectional study.	≈ 65 years *n* = 42 38 % female MMSE: healthy older adults: 28.3 ± 1.3 (*n* = 21) aMCI: 26.7 ± 2.5 (*n* = 21).	**Sleep:** Polysomnography.	Aβ_42_ and Aβ_40,_ Aβ_42/40_ (ELISA).	aMCI, but not healthy older subjects, showed a significant association between ↑ disrupted slow-wave sleep and ↑ Aβ_42._
Cook et al., [56]	African Americans Fighting Alzheimer's in Midlife (AA-FAiM) study. US. Cross-sectional and longitudinal study.	baseline age: 63.2 ± 8.51 years, *n* = 147 69.4 % female Cognitively unimpaired.	**Sleep:** Medical Outcomes Study Sleep Scale (sleep duration) and Epworth Sleepiness Scale (daytime sleepiness).	Aβ_42_ and Aβ_40,_ Aβ_42/40_ (liquid chromatography-tandem mass spectrometry: LC-MS/MS).	Neither self-reported sleep duration nor daytime sleepiness was associated with Aβ_40_, Aβ_42_, or Aβ_42/40_ cross-sectionally or longitudinally.
Benedict et al., (2015) [57]	The Uppsala Longitudinal Study of Adult Men (ULSAM), Sweden. Longitudinal study.	≈ 71 years, *n* = 1029, 0 % female	**Sleep:** Three sleep-related questions answered “no” or “yes”: Do you have difficulties falling asleep at night? Do you often wake up in the early hours, unable to get back to sleep? and Do you take sleeping pills more than 3 times per week? Answering “yes” considered as a sleep disturbance (binary variable).	Aβ_40_, Aβ_42_ (ELISA)	No significant differences were found between reports of sleep disturbance at age 70 years and Aβ_42_ and Aβ_40_ measured at ages 70, 77, and 82 years.
Lysen et al., 2024 [58]	Rotterdam Study cohort. Netherlands. Cross-sectional study.	72 ± 8 years, *n* = 4712 (total sample), *n* = 849 (actigraphy sample) 57 % female.	**Sleep:** Pittsburgh Sleep Quality Index (PSQI) and actigraphy.	Aβ_40_, Aβ_42,_ total-tau and NfL (SiMoA).	No associations of self-rated sleep, actigraphy-estimated sleep and 24-hour activity rhythms with NfL, except for a non-linear association of self-rated time in bed with NfL. No associations with sleep parameters and Aβ_40_, Aβ_42_, and total-tau.

**Abbreviations**: aMCI; amnestic Mild Cognitive Impairment, Aβ; amyloid-β, ELISA; Enzyme-Linked Immunosorbent Assay, GFAP; Glial Fibrillary Acid Protein, LC-MS; liquid chromatography - mass spectrometry, MMSE; Mini Mental State Examination, NfL; Neurofilament Light Chain, PSQI; Pittsburgh Sleep Quality Index, RCT; randomised Controlled trial. SiMoA; Single Molecule Array.

**Table 4:** Summary of studies reporting associations between alcohol consumption, smoking and social isolation and plasma biomarkers of Alzheimer's disease.

Reference	Study (country)	Age, n, gender, cognitive information	Lifestyle factor	Plasma biomarkers investigated and method used	Main findings relating to plasma biomarkers
Hayden et al., 2024 [59]	Action for Health in Diabetes (Look AHEAD) cohort: US. Cross sectional and longitudinal observational study.	61.4 ± 6.2 years *n* = 779 56.2 % female.	**Alcohol and smoking:** No alcohol Alcohol < 21 oz/wk Alcohol ≥21 oz/wk Smoking: never, past and current smoker.	Aβ_42_, Aβ_40_, Aβ_42/40,_ p-tau 181, GFAP, and NfL (SiMoA).	In overweight/obese subjects with type 2 diabetes ↑ alcohol at baseline ↑ baseline GFAP. Smoking ↑Aβ_42_ and ↑ Aβ_40_ over time (≈ 8–13 years) compared to never smoking.
Requena-Ocaña et al., 2023 [60]	Substance use disorder patients from the Centro Provincial de Drogodependencias Málaga. Spain. Cross-sectional study.	41.37 ± 12.40 years, *n* = 60 16.7 % female.	**Alcohol:** Alcohol addiction-related variables.	NfL (SiMoA).	↑ NfL correlated with age at first-time alcohol use, age at alcohol use disorder diagnosis, and length of alcohol use disorder diagnosis.
van der Velpen et al., 2024 [61]	Rotterdam Study cohort. Netherlands. Cross-sectional study.	71.5 ± 7.3 years n = 4099, 57.0 % female MMSE: median 28.0 [IQR:27.0–29.0].	**Social isolation**: Loneliness, marital status and perceived social support were assessed during a home interview. Loneliness was measured on the Center for Epidemiological Studies Depression scale (CES-D).	Aβ_42_, Aβ_40_, Aβ_42/40,_ total-tau, and NfL (SiMoA).	Being never married ↑(ln)Aβ_40_ compared to married peers. Being widowed/divorced ↑(ln)total-tau compared to married peers. Loneliness and social support were not associated with biomarkers.

**Abbreviations**: Aβ; amyloid-β, GFAP; ELISA; Enzyme-Linked Immunosorbent Assay, Glial Fibrillary Acid Protein, IQR; interquartile range, MMSE; Mini Mental State Examination, NfL; Neurofilament Light Chain, SiMoA; Single Molecule Array.

### 3.1 Nutrition and plasma biomarkers of Alzheimer's disease

#### 3.1.1 Observational studies reporting positive findings with individual nutrients

In a cross-sectional study of cognitively healthy participants from the Washington Heights/Hamilton Heights Columbia Aging Project (WHICAP) (age: 75.4 ± 6.1 years, *n* = 1219) omega-3 polyunsaturated fatty acid (n-3 PUFA) consumption, evaluated using the 61-item version of Willett's Semiquantitative Food Frequency Questionnaire (SFFQ), was reported to be inversely associated with plasma Aβ_42_ in adjusted multivariable linear regression [14]. This association was independent of *APOE4* status. n-3 PUFA was also inversely associated with plasma Aβ_40_ in the unadjusted model. Consistent with this, there is direct evidence to suggest that n-3 PUFA specifically decreases β and γ secretase activity and increases the protein stability of α-secretase resulting in non-amyloidogenic processing of amyloid precursor protein (APP) [15]. In contrast, omega-6 (n-6) PUFA, monounsaturated fatty acids (MUFA), saturated fatty acids (SFA), β-carotene, vitamin C, vitamin E, vitamin B_12_, folate, and vitamin D were not associated with neither plasma Aβ_42_ nor plasma Aβ_40_ [14]. Furthermore, cross-sectional analysis of data from the Multidomain Alzheimer Prevention Trial (MAPT) comprising dementia-free participants at risk of cognitive decline (age: 76.8 ± 4.5 years, *n* = 475) showed that erythrocyte docosahexaenoic acid (DHA), eicosapentaenoic acid (EPA) and omega-3 index (measures of n-3 PUFA), as well as plasma homocysteine (Hcy) and plasma 25 hydroxyvitamin D were not associated with plasma Aβ_42/40_ [16]_._ Hcy, a risk factor for cognitive decline and AD [17], was the only nutrient found to be positively associated with plasma NfL in this post-hoc analysis of MAPT data. This finding implies that Hcy might be associated with increased neurodegeneration.

#### 3.1.2 Observational studies reporting positive findings with foods or diet

Proteomic profiling of baseline plasma samples from women without cognitive impairment (baseline age: 71.3 ± 3.8 years, *n* = 1528) from the Women's Health Initiative Memory Study (WHIMS) identified a panel of proteins linked to an inflammatory diet [18]. A subset of these proteins were significantly associated with increased odds of incident cognitive impairment over a 14 year follow-up and positively correlated with baseline plasma NfL and Aβ_42/40_ after adjusting for age [18]. It was suggested that the increased plasma Aβ_42/40_ might reflect lower cerebral Aβ load due to beneficial effects of inflammation [18]. Thus, peripheral inflammation might be associated with increased neurodegeneration through Aβ-independent mechanisms. Furthermore, in a cross-sectional study of cognitively unimpaired Aβ-PET(+) participants (age: 65.2 ± 6.9 years, *n* = 129) it was found that mini-nutritional assessment short form (MNA-SF) total scores (higher score better nutrition: scored out of 14) were positively associated with plasma Aβ_42/40_ [19]. MNA-SF total scores and normal nutritional status (MNA-SF scores: 12–14) were also inversely associated with plasma p-tau 181 in Aβ-PET(+) participants [19]. These findings suggest that normal nutritional status might be associated with less AD pathology in subjects at risk of AD. In another cross-sectional analysis using the three-city study cohort comprising dementia-free participants (age: 76.1 ± 5.2 years, *n* = 377) it was shown that lunch glycaemic load (as opposed to other meals) was associated with lower plasma Aβ_42_ and lower Aβ_42_/Aβ_40_ [20]. These findings suggest that high dietary glycaemic load, as a proxy of refined carbohydrate consumption, might be suggestive of increased cerebral Aβ. Glycaemic load was determined in this study using data from the 148-item semi-quantitative Food Frequency Questionnaire and the reported associations were independent of *APOE4* status [20]. Lastly, in cognitively healthy, obese adults (age: 60 ± 7 years, *n* = 111) plasma NfL, GFAP, Aβ_42/40_, p-tau 181, and p-tau 231 levels were reported to significantly change over a 3-hour post-prandial period following the ingestion of a meal in the form of a protein chocolate drink (300ml: 303.8 kcal; carbohydrates 41.8 g of which 19 g sugar; protein 19.0 g; fat 7.6 g; Boost High Protein, Nestlé Health Science, Vevey, Switzerland) compared to a fasting control group [21]. Fluctuations were multi-phasic for most biomarkers exhibiting both increases and decreases in plasma concentrations over the 3-hour study period. The greatest change to baseline occurred for GFAP and p-tau 181 at 120 minutes postprandially. It should be noted that these post-prandial fluctuations were superimposed on dynamic changes in plasma biomarkers in the ‘control’ fasting state [21]. These findings imply that timings of blood collection relative to eating might impact plasma biomarker analysis.

#### 3.1.3 Observational study with negative findings

No associations were found between intake of protein, fibre, or the protein to fibre intake ratio assessed using the Cancer Council of Victoria Food Frequency Questionnaire (CCVFFQ) and plasma Aβ_40_, Aβ_42_ or Aβ_42/40_ cross-sectionally in cognitively normal adults from the Australian Imaging, Biomarkers and Lifestyle (AIBL) study of aging (age: 69.8 ± 6.8 years, *n* = 541) [22].

#### 3.1.4 A nutrition-based intervention study with positive findings

In the TFA-AD trial, a single-centre randomised controlled trial (RCT), it was shown that folic acid supplementation (1.25 mg/day) for 6 months significantly lowered plasma Aβ_40_ and increased Aβ_42/40_ compared to placebo in subjects with AD being treated with donepezil (age: > 55 years, *n* = 121) [23]. There was no significant change in plasma Aβ_42_ in the intervention group compared to placebo. In this RCT plasma Aβ was evaluated as a primary outcome measure. Presenilin 1 (part of the γ secretase complex) mRNA was also significantly lower in the intervention group versus the control group, which suggests reduced processing of APP towards Aβ_40_ production might mediate these effects.

#### 3.1.5 Nutrition-based intervention studies with negative findings

A secondary analysis of the multi-centre Nolan RCT showed that consumption of a nutritional blend (composed of thiamin, riboflavin, niacin, pantothenic acid, pyridoxine, biotin, folic acid, cobalamin, vitamin E, vitamin C, vitamin D, choline, selenium, citrulline, EPA and DHA) for one year had no significant effects over placebo on plasma p-tau 181 and GFAP levels in community-dwelling older adults with self-reporting subjective memory complaints (age 78.1 ± 4.7 years, *n* = 289) [24]. In addition, a different single-centre RCT in older adults without cognitive dysfunction (mean age: 79.0 ± 8.8 years, *n* = 43) investigating the effects of dietary advice to reduce advanced glycation end products alone or in combination with macrophage-activating factor reported no significant differences in plasma Aβ_42/40_ between the 2 intervention groups and placebo [25]. Furthermore, a secondary analysis of the multi-centre MAPT RCT testing a 3-year multidomain intervention, n-3 PUFA supplementation, or both versus placebo showed no significant differences in change in plasma p-tau 181 between the placebo and the 3 intervention groups [26]. The trial population comprised dementia-free older adults (median age: 74 years, IQR: 72–78, *n* = 527) at risk of cognitive decline.

### 3.2 Physical activity and plasma biomarkers of Alzheimer's disease

#### 3.2.1 Observational studies reporting positive findings primarily on plasma NfL

Physical function (measured using grip strength and gait combined) has been negatively associated with plasma NfL cross-sectionally in a sub-group of participants from the Chinese preclinical Alzheimer's disease study (C-PAS) independent of gender [27]. Participants exhibited ranging levels of cognition from normal cognition through to severe AD in this study (age of total study population: 67.9 ± 11.5 years, *n* = 1048 in biomarker sub-analysis) [27]. Physical function was also found to be negatively associated with brain Aβ deposition in women. These findings suggest that physical function might be associated with reduced cerebral amyloidogenesis in women and reduced neurodegeneration *per se*. Consistent with the latter, in a population-based cross-sectional study on dementia-free participants in the baseline analysis of the Multimodal INterventions to delay Dementia and disability in rural China (MIND-China) RCT (age: 69.56 ± 4.47 years, *n* = 1029) non-linear relationships were reported between daily accelerometer-measured sedentary behaviour (SB) and PA time and plasma NfL [28]. Specifically, more daily SB time or less time spent in daily light-intensity physical activity (LPA) and moderate-to-vigorous-intensity physical activity (MVPA) was significantly associated with increased plasma NfL: when SB time was ≥ 8.00 h/day or LPA time was < 5.00 h/day or MVPA time was < 2.00 h/day [28]. In this study it was reported that low-grade inflammation partially mediated the association between SB and PA and NfL [28]. Furthermore, a post-hoc cross-sectional analysis of the MAPT study comprising a subset of dementia-free older adults at risk of cognitive decline (age: 76.8 ± 4.5 years, *n* = 465) showed that people achieving 500–999 metabolic equivalent task (MET)-min/week (≈ 2–4 h/week of brisk walking) and 2000+ MET-min/week (≈ 8 h+/week of brisk walking) of PA had lower plasma (ln)NfL compared to the control PA group (inactive) [29]. Performing at least 90 MET-min/week (≈ 25 min-2 h/week of brisk walking) of PA was associated with a lower probability of having high plasma NfL. Note, PA was not associated with plasma Aβ_42/40_ in this study [29]. In a different cross-sectional post-hoc analysis of the MAPT study (age: 76 ± 5 years, *n* = 452), no significant association was found between being both Aβ+NfL+ in plasma and physical function (measured as gait speed and chair stand time) [30]. However, in longitudinal analyses, Aβ+NfL+ participants had greater annual declines in gait speed when *APOE* ε4 genotype was excluded as a co-variate from the mixed effect-linear model [30]. Collectively, these studies provide evidence that more PA might be associated with less NfL-dependent neurodegeneration.

#### 3.2.2 Observational studies reporting positive findings on AΒ

Studies investigating the association of PA with plasma Aβ largely report an inverse association to date [[31], [32], [33]], with only one study demonstrating a positive association [34]. However, the literature is inconsistent regarding the species of Aβ that is lower. Such inverse relationships might potentially reflect altered peripheral metabolism of Aβ (increased clearance or reduced production). The first study to show an inverse association of PA with plasma Aβ was a cross-sectional study using AIBL data [31]. In this study it was shown that high PA levels measured using the International Physical Activity Questionnaire (IPAQ) were associated with lower plasma Aβ_42/40_ in cognitively normal participants (age: 69.6 ± 6.8 years, *n* = 546) in *APOE* ε4 non-carriers [31]. No correlation was observed between PA and plasma Aβ_40_ and Aβ_42_. Conversely, lower levels of cerebral Aβ were observed in higher exercising *APOE* ɛ4 carriers, but not in *APOE* ɛ4 non-carriers. In a more recent, cross-sectional study of cognitively normal older adults (age: 74.0 ± 5.5 years, *n* = 143) from the AIBL study it was shown that higher levels of PA also measured using the IPAQ were associated with lower plasma Aβ_40_ and Aβ_42_. These associations were specific for *APOE* ε4 non-carriers as well as Aβ-PET(-) participants [32]. Plasma Aβ_42/40_ was not analysed as a variable in this study. High PA also exhibited a trend-level association with lower cerebral amyloid levels, but only in individuals with high cerebral amyloidosis [32]. It was proposed that differences in cohort size and the use of high-sensitivity assays in the more recent AIBL study might account for these discrepancies in findings [32]. Furthermore, longitudinal data from the population-based Cardiovascular Health Study (CHS) cognition study, showed that in cognitively normal older adults (age: 79.51 ± 3.15 years, *n* = 149) PA measured using the modified Minnesota Leisure‐Time Activities Questionnaire (MLTAQ) was associated with lower plasma Aβ_42_, 9 and 13 years later, as well as with lower plasma Aβ_42/40_ 13 years on [33]. Plasma Aβ_40_ did not show any significant over time changes. In contrast to the aforementioned studies, in a population-based cross-sectional study on dementia-free participants (age: ≥ 65 years, *n* = 325) who completed the Kasama Study health check in 2019 in Japan it was demonstrated that plasma Aβ_42_ was significantly higher in the group regularly performing dual task exercise (involving concurrent cognitive input) compared to the non-exercise group [34]. Plasma Aβ_42_ was also higher in the group performing single task exercises (minus cognitive input) compared to the non-exercise group, but the results didn't reach statistical significance [34]. The authors suggested that these findings might reflect reduced Aβ in the brain [34].

#### 3.2.3 Physical activity-based intervention studies with positive findings

In a pilot study within the FIT-AD trial, effect sizes for within-group 6-month differences were observed for plasma p-tau 181 in the group allocated to stretching exercise (d = 0.43 [−0.33, 1.19]) and plasma total-tau in the cycling (aerobic) exercise group (d =−0.35 [−0.87, 0.17]) [35]. This represents an increase in p-tau 181 in the stretching group and a decrease in total tau in the cycling group. There was however, a trend towards a small increase in plasma p-tau181 in the cycling group. From these findings the authors suggest that aerobic exercise may have a moderate effect size on reducing or slowing down the increase of plasma p-tau 181. This trial population comprised community-dwelling older adults (age: 77.6 ± 6.99 years, *n* = 26) with mild-to-moderate AD. Participants were allocated to moderate-intensity cycling or low-intensity stretching for 20–50 min, 3 times a week for 6 months [35]. In another RCT, sedentary dementia-free elderly participants (age: ≈ 74 years: *n* = 27) were subjected to a 12 week intervention to examine the effects of dual-task training compared to a control single-task training group [36]. Participants were subjected to 1 hour of exercise training 3 times a week. Plasma Aβ_42/40_ was found to decrease in both groups following the training, although there was no significant difference between groups in plasma Aβ_42/40_ [36]. These findings are consistent with the trend towards an inverse relationship with PA and plasma Aβ seen in the observational studies.

#### 3.2.4 Physical activity-based intervention studies with negative findings

No change in plasma levels of p-tau 181, Aβ_40_, Aβ_42_, Aβ_42/40_, GFAP, or NfL pre- to post-intervention were reported in a secondary analysis of the Intense Physical Activity and Cognition (IPAC) study [37]. The IPAC study was a single-centre RCT including community-dwelling cognitively unimpaired older adults (age: 69.1 ± 5.2 years, *n* = 99) where participants were allocated to one of three groups: an inactive control group, or to a moderate or high intensity exercise group where they cycled twice weekly for six months. In another study using an RCT design, cognitively normal older adults with prediabetes or newly diagnosed type 2 diabetes mellitus (age: ≈ 70 years, *n* = 28) completed 6 months of aerobic exercise or stretching (control) to examine the effects of PA on cognition, insulin sensitivity and cardiorespiratory fitness [38]. Aerobic exercise was performed 4 days a week for 45–60 min per session. In exploratory analysis examining the mechanism of the effects of PA on cognition, there was found to be a non-significant trend towards less plasma Aβ_42_ in the aerobic exercise group relative to the control group [38].

### 3.3 Sleep and plasma biomarkers of Alzheimer's disease

#### 3.3.1 Sleep studies reporting positive findings with objective sleep metrics

In a cross-sectional population-based study in dementia-free people (age ≥ 60 years, *n* = 841), long sleep duration (> 8 h versus normal sleep: 6–8 h) measured objectively was associated with lower Aβ_42_/Aβ_40_ and higher plasma Aβ_40_ and total-tau [39]. Both long and short sleep durations have been associated with cognitive decline [40]. No associations between long sleep duration and Aβ_42_ nor NfL were reported. Increased plasma total tau even without concurrent increases in plasma NfL is still suggestive of neurodegeneration as total tau and NfL capture different aspects of neurodegeneration [41]. In this study, short sleep duration (≤ 6 versus normal sleep: 6–8 hours) was not significantly associated with plasma biomarkers for AD or neurodegeneration [39]. In the Presymptomatic Evaluation of Experimental or Novel Treatments for AD (PREVENT‐AD) cohort comprising people at risk of AD (age: 68.25 ± 5.41 years, *n* = 203) it was shown that day-to day sleep variability (measured over a week) was associated with AD biomarkers cross-sectionally [42]. Specifically, higher variability of both objectively-measured sleep duration and night-time activity count (measure of fragmented sleep) were associated with higher levels of plasma p-tau 231/Aβ_42_ (higher ratio associated with AD) [42]._._ Furthermore, higher plasma p‐tau 231/Aβ_42_ was found to be associated with higher sleep duration variability in *APOE4* carriers. Analysis of single biomarkers in the same study, showed that higher plasma p‐tau 181 and p‐tau 231 were associated with higher sleep duration variability and higher plasma p‐tau 231 was also associated with higher night-time activity count variability [42]. These findings suggest that unstable sleep promotes AD pathology or that AD neuropathology disrupts sleep.

#### 3.3.2 Sleep studies reporting positive findings with self-reported sleep metrics

In a population-based cross-sectional study of cognitively normal participants (age: 57.4 ± 9.7 years, *n* = 1459), sleep quality was reported to be negatively associated with the log-transformed plasma Aβ_40_ [43]. Furthermore, the Pittsburgh Sleep Quality Index (PSQI: scored 0–21 with higher scores meaning poorer sleep) was positively associated with the log-transformed plasma Aβ_40_ and negatively associated with plasma Aβ_42_/Aβ_40_ [43]. These associations were specific for *APOE* ε4 non-carriers. Hence, this study suggests that poor sleep might be associated with altered Aβ metabolism, resulting in more Aβ_40_. In another cross-sectional biomarker study, it was reported that in Aβ-PET(+) adults (age: 64.4 ± 7.8 years, *n* = 335) falling asleep at ≥ 22:00 to ≤ 23:00 was associated with greater levels of plasma Aβ_42_ and Aβ_42/40_ [44]. Elevated plasma Aβ_42/40_ was also associated with high sleep efficiency value, no/mild daytime dysfunction and PSQI score ≤ 5 (good sleep quality) [44]. Furthermore, higher plasma p-tau 181 was associated with sleep latency > 30 min in Aβ-PET(+) adults and moderate/severe sleep disturbance in Aβ-PET(-) adults [44]. These findings are consistent with good sleep quality being associated with less AD pathology. In a different cross-sectional study comprising cognitively normal older adults and people with amnestic MCI (aMCI) (age: 69.68 ± 6.84 years, *n* = 305) it was reported that PSQI score was positively associated with plasma Aβ_42_ in the aMCI group only [45]. Additionally, a higher PSQI score was associated with higher plasma Aβ_42_/Aβ_40_ in both the cognitively normal and aMCI groups. The association between PSQI score and plasma Aβ_42_/Aβ_40_ was stronger in individuals with aMCI relative to the cognitively normal subjects [45]. This suggest that poor sleep is associated with higher levels of plasma Aβ.

There is some evidence to suggest that metabolites associated with AD are cleared from the plasma overnight under normal sleep conditions [46,47]. In a small study examining the associations of sleep duration and fragmentation on plasma Aβ in psychiatrists on-call (age: 34 ± 4.3 years, *n* = 17), it was shown that this physiological reduction in overnight plasma Aβ_42_ decreased with increasing number of sleep interruptions ≤ 15 min [48]. The reduction in overnight plasma Aβ_42_ diminished by 5.4 % per sleep interruption. This study included plasma Aβ data from a maximum of 8 consecutive on-call nights per participant [48].

#### 3.3.3 Sleep deprivation studies – acute effects of sleep loss

Increased morning plasma Aβ_40_ and decreased Aβ_42_/Aβ_40_ was reported in a small single-arm trial on participants exhibiting normal cognitive function (age: 27.3 ± 3.4 years, *n* = 20) who underwent 24  hours of total sleep deprivation [49]. These changes were reversed after sleep recovery. No sleep-related change was observed for Aβ_42_ in this study [49]. In a small cross-over study in healthy young men (age: 22.3 ± 0.5 years, *n* = 15) it was reported that in response to complete overnight sleep loss, the evening to morning change in average plasma total-tau levels increased compared to normal sleep [50]. However, no changes were seen for Aβ_40_, Aβ_42_, NfL or GFAP [50]. In another small cross-over study with cognitively normal participants (age: ≈ 48 years, *n* = 5), it was shown that one night of 36 h of sleep deprivation (versus normal sleep) increased Aβ_40_ and Aβ_42_, total-tau (threonine 181 and 217), and p-tau 181 in CSF [51]. Accordingly, increased neuronal activity during wakefulness is thought to enhance cerebral Aβ production and during sleep metabolites including Aβ are thought to be cleared from the brain [52,53]. Moreover, in the study by Liu et al., 2023, Aβ_40_ and Aβ_42_, total-tau and p-tau 181 were found to be conversely decreased in plasma [51]. These findings were attributed to reduced brain clearance of the AD associated metabolites inferred from a concomitant CSF/plasma albumin ratio decrease associated with sleep deprivation. In AD, brain drainage of Aβ is reduced by as much as 30 % [47]. Considering that the participants in Liu et al., 2023 are older than the participants in the previously described sleep deprivation studies it is conceivable that drainage through the blood brain barrier (BBB) might be more impaired [47].

#### 3.3.4 Sleep cycle stages

Over-night blood sampling of healthy volunteers (age: 39.8 ± 16.0 years, *n* = 60) has shown that slow-wave oscillations (deep sleep) are correlated with higher plasma Aβ_40_ and Aβ_42_, whereas rapid eye movement (REM) sleep is correlated with decreased plasma Aβ_40_ and Aβ_42_ [54]. This might be due to changes in Aβ metabolism according to sleep cycle stages. Moreover, disrupted slow wave sleep has been associated with increased plasma Aβ_42_ in aMCI, but not in healthy older adults (age ≈ 65 years, *n* = 42) [55].

#### 3.3.5 Sleep studies with negative findings

Data from the African Americans Fighting Alzheimer's in Midlife study (AA-FAiM) showed that neither self-reported sleep duration nor daytime sleepiness was associated with plasma Aβ_40_, Aβ_42_, or Aβ_42/40_ cross-sectionally or longitudinally in cognitively unimpaired older adults (baseline age: 63.2 ± 8.51 years, *n* = 147) [56]. In another study, no significant differences were found between reports of sleep disturbance at age 70 years and plasma levels of Aβ_42_ and Aβ_40_ measured at ages 70, 77, and 82 years (age: ≈ 71 years, *n* = 1029) [57]. Furthermore, a study using the Rotterdam cohort reported no cross-sectional associations between self-rated sleep, actigraphy-estimated sleep and 24-hour activity rhythms and NfL, Aβ_40_, Aβ_42_, and total-tau in dementia-free subjects (age: 72 ± 8 years, total sample *n* = 4712, actigraphy sample *n* = 849) [58]. Note, one non-linear association of self-rated time in bed with NfL was however reported in this overall negative study [58].

### 3.4 Alcohol and smoking and plasma biomarkers of Alzheimer's disease

In the Action for Health in Diabetes (Look AHEAD) cohort in overweight/obese subjects with type 2 diabetes (age: 61.4 ± 6.2 years, *n* = 779) higher alcohol consumption at baseline was associated with higher plasma baseline GFAP levels [59]. This is perhaps suggestive of alcohol being associated with increased neuro-inflammation. Plasma NfL concentrations have also been positively correlated with alcohol addiction-related variables including age at first-time alcohol use, age at alcohol use disorder (AUD) diagnosis, and length of AUD diagnosis amongst patients attending outpatient treatment for substance abuse (age: 41.37 ± 12.40 years, *n* = 60) [60]. In terms of smoking, in the Look AHEAD cohort, a history of smoking was associated with an increase in Aβ_42_ and Aβ_40_ levels over time (change over ≈ 8–13 years) compared to never smoking [59].

### 3.5 Social isolation and plasma biomarkers of Alzheimer's disease

To the best of our knowledge only one study, carried out using the Rotterdam cohort, has explored the links between parameters of social isolation and plasma biomarkers of AD [61]. In this cross-sectional analysis, it was found that never being married was associated with higher baseline plasma (ln)Aβ_40_ and being widowed or divorced was associated with higher plasma (ln)total-tau (compared to married peers) in cognitively normal older adults (age: 71.5 ± 7.3 years, *n* = 4099). In the same study, sex-stratified analyses demonstrated that never being married was associated with higher plasma (ln)Aβ_40_, (ln)Aβ_42_, (ln)total-tau and (ln)NfL compared to married peers in male participants. *APOE ε4* carrier status did not modify the associations.

## 4 Discussion

To the best of our knowledge this narrative review is the first to summarise the current literature on the relationships between plasma biomarkers of AD and modifiable lifestyle factors restricted here to nutrition, PA, sleep, alcohol consumption, smoking and social isolation. The review has highlighted that the literature on this topic is scant, thus more research is required before firm conclusions can be drawn. However, from the current evidence it can be tentatively inferred that better nutrition, including limiting lunchtime glycaemic load, is associated with better plasma ATN biomarker status potentially reflecting less cerebral AD pathology. Perhaps counterintuitively, n-3 PUFA was associated with less plasma Aβ_42_ and Aβ_40,_ possibly due to a direct effect on peripheral APP processing [62]. Indeed, there is evidence to suggest that n-3 PUFA reduces cerebral Aβ load in animals [63,64]. Peripheral inflammation [18] and the non-essential amino acid, homocysteine [16], were both associated with increased plasma NfL suggestive of central neurodegenerative processes. In fact, hyperhomocysteinemia induces oxidative stress and inflammation [65,66]. Accordingly, there is a large body of evidence to suggest that inflammation fuels AD pathogenesis playing a significant role in disease progression [67]. Nutrition-based intervention studies composing of nutritional supplements [26,68] or dietary advice (alone or in combination with specific supplementation) [25] have yielded negative results in terms of changes in plasma biomarkers of AD. One RCT has however demonstrated effects of folic acid supplementation on plasma Aβ biomarkers [23]. Folic acid is known to play a positive role in brain health and exhibits a specific homocysteine lowering effect [69]. More research is required to determine the long-term effects of nutrients and dietary patterns on plasma biomarkers of AD and how this relates to central pathology. Of note, the Mediterranean and ketogenic diets have been associated with less cerebral Aβ and the Western diet the converse [70].

PA was found to associate with lower plasma NfL across the studies identified [[27], [28], [29]] and hence is suggestive of less neurodegeneration with evidence suggesting that less inflammation [28], oxidative stress [71] and cardiovascular health might play mediating roles [72]. The majority of studies reporting on the relationships between PA and plasma Aβ demonstrate an inverse relationship [[31], [32], [33]]. Less plasma Aβ is suggestive of increased central Aβ pathology, yet, it is plausible that exercise increases peripheral clearance of Aβ and is not an indicator of enhanced cerebral amyloidogenesis when other variables are controlled for. Plasma Aβ can be degraded by proteases, metabolised in the liver or cleared by the kidneys introducing a variance that is unrelated to brain changes [73]. Evidence regarding the associations between PA and central Aβ is inconclusive [31,32,74,75]. It has been suggested that the effects of PA on AD pathology could be sub-group specific: PA might be more beneficial in women with metabolic disease or depression or in *APOE* ε4 carriers [75]. Further research is required to understand the mechanism through which PA modulates plasma Aβ and how this in turn affects brain pathology. We retrieved one pilot study with positive findings that had focussed on the effects of aerobic exercise on plasma p-tau and a beneficial response in favour of the exercise intervention was reported [35]. Accordingly, more PA has been associated with reduced central tau-PET positivity [76]. Thus, more studies employing plasma p-tau as an outcome measure/dependent variable, a more sensitive biomarker of AD, are required.

The associations of sleep metrics with plasma biomarkers of AD are mixed and there is considerable diversity in the parameters used including sleep duration, sleep quality (latency, efficiency, fragmentation) and daytime dysfunction/sleepiness. Nevertheless, there is an emerging trend that poor sleep is associated with elevated plasma Aβ [43,45,48]. This might reflect diminished plasma clearance [47,48] and not reduced cerebral amyloidogenesis since some evidence suggests that poor sleep is associated with increased cerebral Aβ plaque load [[77], [78], [79], [80]]. It should be borne in mind, however that reverse causality, as an inherent limitation of cross-sectional studies, could mean that higher Aβ levels contribute to poor sleep and fatigue. Future research is required to explore the mechanisms through which sleep, including sleep cycle stages, modulates peripheral Aβ metabolism and how this impacts central pathology. In terms of tauopathy, two studies [42,44] have reported that plasma p-tau is elevated in association with negative sleep variables. Poor sleep has also been associated with increased tau PET positivity in the brain [81,82]. Experimental sleep deprivation studies [49,51,57] further confirm that disrupted sleep alters ATN biomarker profiles in the plasma and CSF, but precise patterns are hard to decipher from the limited data. Thus, more studies are required to examine how AD metabolites are exchanged between the CSF and blood during sleep.

Alcohol consumption, smoking and social isolation have received the least research attention with regard to the lifestyle factors reported on in this review and therefore represent important avenues for future exploration. Higher alcohol intakes and alcohol misuse have been associated with plasma GFAP [59] and NfL [60] respectively. Alcohol use disorder has also been associated with greater odds for increased cerebral Aβ PET positivity in subjects ≥ 65 years old [83]. Furthermore, smoking has been associated with an increase in plasma Aβ temporally [59]. Limited imaging evidence suggests that a history of smoking is associated with increased Aβ PET positivity in the brain in cognitively normal older adults, whilst human autopsy studies have yielded mixed findings regarding the associations between smoking and plaque and tangle count [84]. In terms of social isolation, marital status has been associated with plasma biomarkers of Aβ and neurodegeneration particularly in males [61]. Highlighting potential inter-sex differences in the susceptibility to social health parameters. Data from transgenic mouse models of AD has also demonstrated that social isolation increases cerebral Aβ plaques [85] and the hyper-phosphorylation of tau [86].

A recent multi-centre RCT, the Lifestyle Intervention for Early Alzheimer's Disease trial, including participants with MCI or early AD has shown that 20 weeks of a lifestyle intervention significantly increased plasma Aβ_42/40_ compared to placebo [87]. No significant effects on plasma p-tau 181 or GFAP were reported, although trends in a beneficial direction were evident in the intervention group. The lifestyle intervention consisted of a whole foods minimally processed plant-based diet (vegan), low in harmful fats and low in refined carbohydrates and sweeteners with selected supplements (omega-3 and curcumin, Solgar VM-75 multi-vitamin and mineral tablet, co-enzyme Q10, vitamin C, vitamin B12, magnesium l-threonate, Hericium erinaceus, Flora Super Bifido Plus Probiotic), moderate exercise, stress management techniques and support groups. Thus, modifying more than one lifestyle factor simultaneously might be more efficacious in terms of improving plasma AD biomarker profiles.

The strengths of this narrative review are that it has highlighted the scarcity of reports on lifestyle factors and plasma biomarkers of AD emphasising the need for further research. There are however a number of limitations to this review. Firstly, its narrative nature implies that not every published study was captured despite the implementation of a systematic search method. The studies described used populations varying in ethnicity, age and cognitive status and it is recognised that individuals that volunteer to participate in research studies are of higher educational status and are therefore not representative of the general population. Some studies were also small in participant number and therefore might not have been sufficiently powered to detect changes in plasma biomarkers. Furthermore, caution should be employed when comparing results between studies since different assays were used to measure plasma biomarkers and older studies did not employ new ultra-sensitive techniques. Heterogeneity in the lifestyle factor outcome variables (ie self-reported versus objectively measured/ questionnaire versus biochemical measurement) further hinders comparisons. Lastly, most of the observational studies described were cross-sectional and therefore only provide a snapshot of data and thus cause-and-effect relationships cannot be drawn.

## 5 Conclusion

Despite the potential of modifiable lifestyle factors for preserving cognition [12,13,88], more high-level research, in terms of larger longitudinal studies, is required to determine how such lifestyle factors modulate plasma biomarkers of AD. Future research direction could include subgroup analysis according to variables such as gender, metabolic health and *APOE* status to understand the modifying effects of such parameters on lifestyle-related plasma AD biomarker profiles. Lifestyle intervention RCTs with plasma biomarkers as their primary outcome could also be considered. Modifying/optimising multiple lifestyle factors might also prove more efficacious. Given that lifestyle modifications are generally low-cost and accessible compared to pharmacological treatments such deeper investigation is warranted.

## CRediT authorship contribution statement

**Claudie Hooper:** Writing – original draft. **Nicola Coley:** Writing – review & editing. **Julien Delrieu:** Writing – review & editing, Conceptualization. **Sophie Guyonnet:** Writing – review & editing, Conceptualization.

## Declaration of interests

The authors declare that they have no known competing financial interests or personal relationships that could have appeared to influence the work reported in this paper.

## References

[1] 2023 Alzheimer's disease facts and figures. Alzheimer Dement 2023;19:1598–695. 10.1002/alz.13016. 36918389

[2] Hardy J., Allsop D. (1991). Amyloid deposition as the central event in the aetiology of Alzheimer's disease. Trends Pharmacol Sci.

[3] Hardy J.A., Higgins G.A. (1992). Alzheimer's disease: the amyloid cascade hypothesis. Science.

[4] Jack C.R., Bennett D.A., Blennow K., Carrillo M.C., Dunn B., Haeberlein S.B. (2018). NIA-AA Research Framework: toward a biological definition of Alzheimer's disease. Alzheimer Dement.

[5] Leuzy A., Mattsson-Carlgren N., Palmqvist S., Janelidze S., Dage J.L., Hansson O. (2022). Blood-based biomarkers for Alzheimer's disease. EMBO Mol Med.

[6] Arslan B., Zetterberg H., Ashton N.J. (2024). Blood-based biomarkers in Alzheimer's disease - moving towards a new era of diagnostics. Clin Chem Lab Med.

[7] Janelidze S., Stomrud E., Palmqvist S., Zetterberg H., van Westen D., Jeromin A. (2016). Plasma β-amyloid in Alzheimer's disease and vascular disease. Sci Rep.

[8] Dark H.E., Duggan M.R., Walker K.A. (2024). Plasma biomarkers for Alzheimer's and related dementias: a review and outlook for clinical neuropsychology. Arch Clin Neuropsychol.

[9] Jack C.R., Bennett D.A., Blennow K., Carrillo M.C., Feldman H.H., Frisoni G.B. (2016). A/T/N: an unbiased descriptive classification scheme for Alzheimer disease biomarkers. Neurology.

[10] Leipp F., Vialaret J., Mohaupt P., Coppens S., Jaffuel A., Niehoff A.-C. (2024). Glial fibrillary acidic protein in Alzheimer's disease: a narrative review. Brain Commun.

[11] Yang Z., Sreenivasan K., Toledano Strom E.N., Osse A.M.L., Pasia L.G., Cosme C.G. (2023). Clinical and biological relevance of glial fibrillary acidic protein in Alzheimer's disease. Alzheimer Res Ther.

[12] Livingston G., Huntley J., Sommerlad A., Ames D., Ballard C., Banerjee S. (2020). Dementia prevention, intervention, and care: 2020 report of the Lancet Commission. Lancet.

[13] Livingston G., Huntley J., Liu K.Y., Costafreda S.G., Selbæk G., Alladi S. (2024). Dementia prevention, intervention, and care: 2024 report of the Lancet standing commission. Lancet.

[14] Gu Y., Schupf N., Cosentino S.A., Luchsinger J.A., Scarmeas N. (2012). Nutrient intake and plasma β-amyloid. Neurology.

[15] Grimm M.O.W., Kuchenbecker J., Grösgen S., Burg V.K., Hundsdörfer B., Rothhaar T.L. (2011). Docosahexaenoic acid reduces amyloid beta production via multiple pleiotropic mechanisms. J Biol Chem.

[16] Giudici K.V., de Souto, Barreto P., Guyonnet S., Morley J.E., Nguyen A.D., Aggarwal G. (2023). TNFR-1 and GDF-15 are associated with plasma neurofilament light chain and progranulin among community-dwelling older adults: a secondary analysis of the MAPT study. J Gerontol A Biol Sci Med Sci.

[17] Smith A.D., Refsum H., Bottiglieri T., Fenech M., Hooshmand B., McCaddon A. (2018). Homocysteine and dementia: an international consensus statement. J Alzheimer Dis.

[18] Duggan M.R., Butler L., Peng Z., Daya G.N., Moghekar A., An Y. (2023). Plasma proteins related to inflammatory diet predict future cognitive impairment. Mol Psychiatry.

[19] Chu H., Huang C., Guan Y., Xie F., Chen M., Guo Q. (2024). The associations between nutritional status and physical frailty and Alzheimer's disease plasma biomarkers in older cognitively unimpaired adults with positive of amyloid-β PET. Clin Nutr.

[20] Gentreau M., Raymond M., Samieri C., Chuy V., Féart C., Berticat C. (2022). Dietary glycemic load and plasma amyloid-β biomarkers of Alzheimer's disease. Nutrients.

[21] Huber H., Ashton N.J., Schieren A., Montoliu-Gaya L., Molfetta G.D., Brum W.S. (2023). Levels of Alzheimer's disease blood biomarkers are altered after food intake-A pilot intervention study in healthy adults. Alzheimer Dement.

[22] Fernando W.M.A.D.B., Rainey-Smith S.R., Gardener S.L., Villemagne V.L., Burnham S.C., Macaulay S.L. (2018). Associations of dietary protein and Fiber intake with brain and blood amyloid-β. J Alzheimers Dis.

[23] Chen H., Liu S., Ji L., Wu T., Ji Y., Zhou Y. (2016). Folic acid supplementation mitigates Alzheimer's disease by reducing inflammation: a randomized controlled trial. Mediat Inflamm.

[24] Giudici K.V., de Souto, Barreto P., Guyonnet S., Cantet C., Zetterberg H., Boschat C. (2023). Effect of a 1-year nutritional blend supplementation on plasma p-tau181 and GFAP levels among community-dwelling older adults: a secondary analysis of the Nolan trial. JAR Life.

[25] Uchiyama-Tanaka Y., Yamakage H., Inui T. (2024). The effects of dietary intervention and macrophage-activating factor supplementation on cognitive function in elderly users of outpatient rehabilitation. Nutrients.

[26] Coley N., Zetterberg H., Cantet C., Guyonnet S., Ashton N.J., Vellas B. (2024). Plasma p-tau181 as an outcome and predictor of multidomain intervention effects: a secondary analysis of a randomised, controlled, dementia prevention trial. Lancet Health Longev.

[27] Guo Y., Huang L., Kuang J., Sun T., Zhang X., Tian H. (2024). Physical function is associated with cognitive status, brain amyloid-beta deposition, and blood biomarkers in Chinese Han population. CNS Neurosci Ther.

[28] Wang Y., Han Q., Han X., Dong Y., Mao M., Wang C. (2023). Objectively-measured movement behaviors, systemic low-grade inflammation, and plasma neurofilament light chain in older adults: a population-based study. Immun Age.

[29] Raffin J., Rolland Y., Aggarwal G., Nguyen A.D., Morley J.E., Li Y. (2021). Associations between physical activity, blood-based biomarkers of neurodegeneration, and cognition in healthy older adults: the MAPT Study. J Gerontol A Biol Sci Med Sci.

[30] He L., de Souto, Barreto P., Aggarwal G., Nguyen A.D., Morley J.E., Li Y. (2020). Plasma aβ and neurofilament light chain are associated with cognitive and physical function decline in non-dementia older adults. Alzheimer Res Ther.

[31] Brown B.M., Peiffer J.J., Taddei K., Lui J.K., Laws S.M., Gupta V.B. (2013). Physical activity and amyloid-β plasma and brain levels: results from the Australian Imaging, Biomarkers and Lifestyle Study of ageing. Mol Psychiatry.

[32] Pedrini S., Chatterjee P., Nakamura A., Tegg M., Hone E., Rainey-Smith S.R. (2022). The association between Alzheimer's disease-related markers and physical activity in cognitively normal older adults. Front Aging Neurosci.

[33] Stillman C.M., Lopez O.L., Becker J.T., Kuller L.H., Mehta P.D., Tracy R.P. (2017). Physical activity predicts reduced plasma β amyloid in the Cardiovascular Health Study. Ann Clin Transl Neurol.

[34] Yoon J., Sasaki K., Tateoka K., Arai T., Isoda H., Okura T. (2023). Evaluation of cognitive and physical function among older adults by their physical activity: a cross-sectional Kasama study, Japan. J Alzheimer Dis.

[35] Yu F., Han S.Y., Salisbury D., Pruzin J.J., Geda Y., Caselli R.J. (2022). Feasibility and preliminary effects of exercise interventions on plasma biomarkers of Alzheimer's disease in the FIT-AD trial: a randomized pilot study in older adults with Alzheimer's dementia. Pilot Feasibil Stud.

[36] Yokoyama H., Okazaki K., Imai D., Yamashina Y., Takeda R., Naghavi N. (2015). The effect of cognitive-motor dual-task training on cognitive function and plasma amyloid β peptide 42/40 ratio in healthy elderly persons: a randomized controlled trial. BMC Geriatr.

[37] Sewell K.R., Rainey-Smith S.R., Pedrini S., Peiffer J.J., Sohrabi H.R., Taddei K. (2024). The impact of exercise on blood-based biomarkers of Alzheimer's disease in cognitively unimpaired older adults. Geroscience.

[38] Baker L.D., Frank L.L., Foster-Schubert K., Green P.S., Wilkinson C.W., McTiernan A. (2010). Aerobic exercise improves cognition for older adults with glucose intolerance, a risk factor for Alzheimer's disease. J Alzheimer Dis.

[39] Tang S., Liu R., Ren J., Song L., Dong L., Qin Y. (2024). Association of objective sleep duration with cognition and brain aging biomarkers in older adults. Brain Commun.

[40] Yang Q., Li S., Yang Y., Lin X., Yang M., Tian C. (2024). Prolonged sleep duration as a predictor of cognitive decline: a meta-analysis encompassing 49 cohort studies. Neurosci Biobehav Rev.

[41] Kasuga K., Kikuchi M., Tsukie T., Suzuki K., Ihara R., Iwata A. (2022). Different AT(N) profiles and clinical progression classified by two different N markers using total tau and neurofilament light chain in cerebrospinal fluid. BMJ Neurol Open.

[42] Baril A., Picard C., Labonté A., Sanchez E., Duclos C., Mohammediyan B. (2024). Day-to-day sleep variability with Alzheimer's biomarkers in at-risk elderly. Alzheimer Dement (Amst).

[43] Gao Y., Wei S., Gao F., Gao L., Dang L., Shang S. (2021). Sleep disturbance is associated with higher plasma aβ levels in cognitively normal adults—a population-based cross-sectional study. Front Aging Neurosci.

[44] Chu H., Huang C., Miao Y., Ren C., Guan Y., Xie F. (2023). The association of subjective sleep characteristics and plasma biomarkers of Alzheimer's disease pathology in older cognitively unimpaired adults with higher amyloid-β burden. J Neurol.

[45] Liu Y., Chen L., Huang S., Zhang C., Lv Z., Luo J. (2020). Subjective sleep quality in amnestic mild cognitive impairment elderly and its possible relationship with plasma amyloid-β. Front Neurosci.

[46] della Monica C., Revell V., Atzori G., Laban R., Skene S.S., Heslegrave A. (2024). P-tau217 and other blood biomarkers of dementia: variation with time of day. Transl Psychiatry.

[47] Cheng Y., Tian D.-Y., Wang Y.-J. (2020). Peripheral clearance of brain-derived aβ in Alzheimer's disease: pathophysiology and therapeutic perspectives. Transl Neurodegener.

[48] Grimmer T., Laub T., Hapfelmeier A., Eisele T., Fatke B., Hölzle P. (2020). The overnight reduction of amyloid β 1-42 plasma levels is diminished by the extent of sleep fragmentation, sAPP-β, and APOE ε4 in psychiatrists on call. Alzheimer Dement.

[49] Wei M., Zhao B., Huo K., Deng Y., Shang S., Liu J. (2017). Sleep deprivation induced plasma amyloid-β transport disturbance in healthy young adults. J Alzheimer Dis.

[50] Benedict C., Blennow K., Zetterberg H., Cedernaes J. (2020). Effects of acute sleep loss on diurnal plasma dynamics of CNS health biomarkers in young men. Neurology.

[51] Liu H., Barthélemy N.R., Ovod V., Bollinger J.G., He Y., Chahin S.L. (2023). Acute sleep loss decreases CSF-to-blood clearance of Alzheimer's disease biomarkers. Alzheimer Dement.

[52] Musiek E.S., Xiong D.D., Holtzman D.M. (2015). Sleep, circadian rhythms, and the pathogenesis of Alzheimer disease. Exp Mol Med.

[53] Harenbrock J., Holling H., Reid G., Koychev I. (2023). A meta-analysis of the relationship between sleep and β-amyloid biomarkers in Alzheimer's disease. Biomark Neuropsychiatry.

[54] Rosenblum Y., Pereira M., Stange O., Weber F.D., Bovy L., Tzioridou S. (2024). Divergent associations of slow-wave sleep versus rapid eye movement sleep with plasma amyloid-beta. Ann Neurol.

[55] Sanchez-Espinosa M.P., Atienza M., Cantero J.L. (2014). Sleep deficits in mild cognitive impairment are related to increased levels of plasma amyloid-β and cortical thinning. NeuroImage.

[56] Cook J.D., A M, Dt P., D N, R LK, L D (2024). Associations of sleep duration and daytime sleepiness with plasma amyloid beta and cognitive performance in cognitively unimpaired, middle-aged and older African Americans. Sleep.

[57] Benedict C., Byberg L., Cedernaes J., Hogenkamp P.S., Giedratis V., Kilander L. (2015). Self-reported sleep disturbance is associated with Alzheimer's disease risk in men. Alzheimer Dement.

[58] Lysen T.S., Ikram M.A., Ghanbari M., Luik A.I. (2020). Sleep, 24-h activity rhythms, and plasma markers of neurodegenerative disease. Sci Rep.

[59] Hayden K.M., Mielke M.M., Evans J.K., Neiberg R., Molina-Henry D., Culkin M. (2024). Association between modifiable risk factors and levels of blood-based biomarkers of Alzheimer's and related dementias in the look AHEAD cohort. JAR Life.

[60] Requena-Ocaña N., Araos P., Serrano-Castro P.J., Flores-López M., García-Marchena N., Oliver-Martos B. (2023). Plasma concentrations of neurofilament light chain protein and brain-derived neurotrophic factor as consistent biomarkers of cognitive impairment in alcohol use disorder. Int J Mol Sci.

[61] van der Velpen I.F., Yaqub A., Vernooij M.W., Perry M., Vernooij-Dassen M.J.F., Ghanbari M. (2024). Sex-differences in the association of social health and marital status with blood-based immune and neurodegeneration markers in a cohort of community-dwelling older adults. Brain Behav Immun.

[62] Grimm M.O.W., Mett J., Hartmann T. (2016). The impact of vitamin E and other fat-soluble vitamins on Alzheimer´s disease. Int J Mol Sci.

[63] Perez S.E., Berg B.M., Moore K.A., He B., Counts S.E., Fritz J.J. (2010). DHA diet reduces AD pathology in young APPswe/PS1 Delta E9 transgenic mice: possible gender effects. J Neurosci Res.

[64] Oksman M., Iivonen H., Hogyes E., Amtul Z., Penke B., Leenders I. (2006). Impact of different saturated fatty acid, polyunsaturated fatty acid and cholesterol containing diets on beta-amyloid accumulation in APP/PS1 transgenic mice. Neurobiol Dis.

[65] Kennedy D.O. (2016). B vitamins and the brain: mechanisms, dose and efficacy—a review. Nutrients.

[66] González-Lamuño D., Arrieta-Blanco F.J., Fuentes E.D., Forga-Visa M.T., Morales-Conejo M., Peña-Quintana L. (2024). Hyperhomocysteinemia in adult patients: a treatable metabolic condition. Nutrients.

[67] Kinney J.W., Bemiller S.M., Murtishaw A.S., Leisgang A.M., Salazar A.M., Lamb B.T. (2018). Inflammation as a central mechanism in Alzheimer's disease. Alzheimer Dement (NY).

[68] Giudici K.V., de Souto, Barreto P., Guyonnet S., Cantet C., Zetterberg H., Boschat C. (2023). Effect of a 1-year nutritional blend supplementation on plasma p-tau181 and GFAP levels among community-dwelling older adults: a secondary analysis of the Nolan trial. JAR Life.

[69] Ekundayo B.E., Adewale O.B., Obafemi T.O. Neuroprotective effects of folic acid: a review. J Diet Suppl n.d.;0:1–19. 10.1080/19390211.2024.2436842. 39648692

[70] Díaz G., Lengele L., Sourdet S., Soriano G., de Souto, Barreto P. (2022). Nutrients and amyloid β status in the brain: a narrative review. Age Res Rev.

[71] Li B., Liang F., Ding X., Yan Q., Zhao Y., Zhang X. (2019). Interval and continuous exercise overcome memory deficits related to β-amyloid accumulation through modulating mitochondrial dynamics. Behav Brain Res.

[72] Stojanovic M., Schindler S.E., Morris J.C., Head D. (2023). Effect of exercise engagement and cardiovascular risk on neuronal injury. Alzheimer Dement.

[73] Blennow K., Zetterberg H. (2018). Biomarkers for Alzheimer's disease: current status and prospects for the future. J Intern Med.

[74] de Souto Barreto P., Andrieu S., Rolland Y. (2015). Physical activity and β-amyloid brain levels in humans: a systematic review. J Prev Alzheimer Dis.

[75] Raffin J. Does physical exercise modify the pathophysiology of Alzheimer's Disease in older persons? JAR Life n.d.;13:77–81. 10.14283/jarlife.2024.11. PMC11129780 38803456

[76] Gonzales M.M., Kojis D., Spartano N.L., Thibault E.G., DeCarli C.S., El Fakhri G. (2024). Associations of physical activity engagement with cerebral amyloid-β and tau from midlife. J Alzheimer Dis.

[77] Spira A.P., Gamaldo A.A., An Y., Wu M.N., Simonsick E.M., Bilgel M. (2013). Self-reported sleep and β-amyloid deposition in community-dwelling older adults. JAMA Neurol.

[78] Ettore E., Bakardjian H., Solé M., Levy Nogueira M., Habert M.-O., Gabelle A. (2019). Relationships between objectives sleep parameters and brain amyloid load in subjects at risk for Alzheimer's disease: the INSIGHT-preAD Study. Sleep.

[79] Brown B.M., Rainey-Smith S.R., Villemagne V.L., Weinborn M., Bucks R.S., Sohrabi H.R. (2016). The relationship between sleep quality and brain amyloid burden. Sleep.

[80] Hooper C., De Souto, Barreto P., Coley N., Cesari M., Payoux P., Salabert A.S. (2017). Cross-sectional associations of fatigue with cerebral β-amyloid in older adults at risk of dementia. Front Med (Lausanne).

[81] Fernandes M., Chiaravalloti A., Cassetta E., Placidi F., Mercuri N.B., Liguori C. (2024). Sleep fragmentation and Sleep-wake cycle dysregulation are associated with cerebral tau burden in patients with mild cognitive impairment due to Alzheimer's Disease: a case series. J Alzheimer Dis Rep.

[82] Kim R.T., Zhou L., Li Y., Krieger A.C., Nordvig A.S., Butler T. (2024). Impaired sleep is associated with tau deposition on 18F-flortaucipir PET and accelerated cognitive decline, accounting for medications that affect sleep. J Neurol Sci.

[83] Essa H., Ali H.M., Min P.H., Ali D.N., Lowe V., Petersen R.C. (2024). Impact of alcohol use disorder on cognition in correlation with aging: a community-based retrospective cohort study. Alcohol Alcohol.

[84] Durazzo T.C., Mattsson N., Weiner M.W. (2014). Smoking and increased Alzheimer's disease risk: a review of potential mechanisms. Alzheimer Dement.

[85] Dong H., Goico B., Martin M., Csernansky C.A., Bertchume A., Csernansky J.G. (2004). Modulation of hippocampal cell proliferation, memory, and amyloid plaque deposition in APPsw (Tg2576) mutant mice by isolation stress. Neuroscience.

[86] Ren Q.-G., Gong W.-G., Wang Y.-J., Zhou Q.-D., Zhang Z.-J. (2015). Citalopram attenuates tau hyperphosphorylation and spatial memory deficit induced by social isolation rearing in middle-aged rats. J Mol Neurosci.

[87] Ornish D., Madison C., Kivipelto M., Kemp C., McCulloch C.E., Galasko D. (2024). Effects of intensive lifestyle changes on the progression of mild cognitive impairment or early dementia due to Alzheimer's disease: a randomized, controlled clinical trial. Alzheimer Res Therapy.

[88] Ko Y., Chye S.M. (2020). Lifestyle intervention to prevent Alzheimer's disease. Rev Neurosci.

